# Determinants of early initiation of breastfeeding in Ghana: a population-based cross-sectional study using the 2014 Demographic and Health Survey data

**DOI:** 10.1186/s12884-020-03308-w

**Published:** 2020-10-19

**Authors:** Abdul-Aziz Seidu, Edward Kwabena Ameyaw, Bright Opoku Ahinkorah, Freda Bonsu

**Affiliations:** 1 Department of Population and Health, University of Cape Coast, Cape Coast, Ghana; 2 College of Public Health, Medical and Veterinary Sciences, James Cook University, Townsville, Queensland Australia; 3 School of Public Health, Faculty of Health, University of Technology Sydney, Sydney, Australia; 4 Asutifi South District Health Directorate, Hwidiem, Ghana

**Keywords:** Early initiation, Breastfeeding, Newborn health, Ghana

## Abstract

**Background:**

The World Health Organisation (WHO) recommends that breastfeeding should be initiated within the first hour of delivery followed by exclusive breastfeeding up to 6 months. This study examined the determinants of early initiation of breastfeeding in Ghana using data from the 2014 Ghana Demographic and Health Survey.

**Methods:**

A sample size of 4219 was used for the study. Descriptive statistics was conducted to ascertain the proportion of children who had early initiation of breastfeeding after which binary logistic regression analysis was carried out. Results were presented using frequencies, percentages, unadjusted and adjusted odds ratios. Statistical significance was pegged at p<0.05.

**Results:**

Children of first birth order [AOR = 0.71, CI = 0.61–0.84], those who were delivered by non-professionals [AOR = 0.51, CI = 0.30–0.88] and those whose mothers were Traditionalists [AOR = 0.65, CI = 0.46–0.92] and Mole-Dagbanis [AOR = 0.69, CI = 0.54–0.89] were less likely to go through early initiation of breastfeeding compared to those of 2–4 birth order, those who were delivered by health professionals, those whose mothers were Christians and Akan, respectively. Conversely, children born to mothers who read newspaper/magazine at least once a week were more likely to go through early initiation of breastfeeding, compared to those who never read newspaper/magazine [AOR = 1.40, CI = 1.01–1.95]. Children born to mothers who watched television less than once a week were more likely to go through early initiation of breastfeeding compared to those who watched television at least once a week [AOR = 1.40, CI = 1.01–1.95]. Finally, women from the Northern [AOR = 2.40, CI = [1.77–3.26] and Upper East regions [AOR = 2.57, CI = [1.86–3.56] practiced early initiation of breastfeeding compared to those from the Ashanti region.

**Conclusions:**

Empowering healthcare providers to be consistent in early breastfeeding initiation advocacy and effective community engagement on the need to embrace and practice early initiation of breastfeeding can improve the situation.

## Background

Breastfeeding is essential for the growth and development of newborns by providing vital nutrients [1]. The World Health Organisation (WHO) recommends that breastfeeding should be initiated within the first hour of delivery followed by exclusive breastfeeding up to 6 months [2]. Early initiation of breastfeeding is defined as the initiation of breast milk feeding within 1 hour after delivery [3]. Early initiation of breastfeeding has several health benefits such as increased ability of the immune system to resist infections, reduction in the risk of diarrhea, and increased survival rate of children [4]. According to Mugadza, Zvinavashe, Gumbo and Pedersen [5], early initiation of breastfeeding reduces neonatal mortality by 33%. On the other hand, Berkat and Sutan [6] found that late initiation of breastfeeding leads to high neonatal morbidity and mortality.

This is affirmed by some recent systematic reviews and meta-analysis. For instance, Smith and colleagues reported that infants whose breastfeeding initiation occur between 2 and 23 hours after birth have 33% increased risk of neonatal mortality compared with those whose initiation occurs in less than 1 hour [7]. Prospective analysis of pooled randomised trials from Ghana, India, and Tanzania also revealed 1.79 increased likelihood of mortality among infants whose breastfeeding initiation occurred between 2 and 23 hours compared to those initiated within the first hour [8].

Despite WHO’s recommendation that every newborn should be given breast milk within 1 hour after delivery, a lot of mothers in low-and middle-income countries do not practice early initiation of breastfeeding [9–11]. In Ghana, a little over half (56%) of children are breastfed within 1 hour of birth [12]. This situation is worrisome considering the fact that one in ten children (12%) had diarrhoea in 2014, with a neonatal mortality rate of 29 deaths per 1000 live births. Additionally, infant mortality rate stood at 41 deaths per 1000 live births with under-5 mortality rate of 60 deaths per 1000 live births [12].

Several studies in low and middle-income countries, have identified mother’s age, birth order, preceding birth interval, place of delivery, mode of delivery, gender of the child, wealth index, residence and region as determinants of early initiation of breastfeeding [13–15]. A study that was conducted in one of the Districts in Ghana used qualitative approach to explore why women initiate breast-feeding early or late, who gives advice about initiation and what foods or fluids are given to babies when breastfeeding initiation is late [16]. To the best of our knowledge, it appears there is no study in Ghana that has looked at the determinants of early initiation of breastfeeding using nationally representative data. This study, therefore, examined the determinants of early initiation of breastfeeding in Ghana using data from the 2014 Ghana Demographic and Health Survey (GDHS). Findings from the study will be useful to policymakers, health care providers, and stakeholders in improving the practice of early initiation of breastfeeding in the country.

## Methods

### Description of the survey and sampling

We used data from the child recode file of the 2014 version of the Ghana Demographic and Health Survey (GDHS). The GDHS is a nationwide survey which covers all the erstwhile ten regions and is conducted every 5 years [12]. The survey is conducted by the Ghana Statistical Service and the Ghana Health Service with Inner City Fund(ICF) International giving technical support through the MEASURE DHS Program. The survey adopts a two-stage sampling design. The first stage involves the selection of clusters consisting of enumeration areas delineated for the Population and Housing Census preceding the survey [12]. The second stage involved the selection of households from each cluster. A total of 9396 women (97.3% response rate) were interviewed for the survey. The survey provides a complete birth history of women and their children. Further details of the methodology can be found in the final report [12]. Actual sample of mothers with children having complete cases on the variables used in this study were 4219 children. Permission to use the dataset was obtained from MEASURE DHS. Dataset is available to the public at www.measuredhs.org. We relied on the “Strengthening the Reporting of Observational Studies in Epidemiology” (STROBE) statement in writing the manuscript.

### Definition of variables

The outcome variable was derived from the question “How long after birth did you first put (NAME) to the breast?” Responses were recorded in number of hours or days [14]. Our outcome variable “early initiation breastfeeding” was defined as initiation of breastfeeding within 1 hour of birth and was expressed as a dichotomous variable with category 1 for initiation of breastfeeding within 1 hour (early) and category 0 for initiation of breastfeeding after 1 hour (late). ﻿﻿The independent variables were chosen based on previous studies and availability in the dataset [1, 13]. Some of the variables were recoded while others were adopted as reported in the 2014 GDHS. These included sex of child, size at birth, birth order, twin status, type of delivery assistance, place child was delivered (child factors). Others were mother’s age, marital status, religion, ethnicity, educational level, working status, frequency of listening to radio, frequency of listening to radio, frequency of watching television, number of ANC visits (maternal factors) and region , place of residence and wealth index (community/household factors) (see Table 1).

**Table 1:** Distribution of early initiation of breastfeeding across the child, maternal and community/household level characteristics of women

Variable	Initiation of breastfeeding (***n*** = 4219)	
	After 1 hour n (%) 1869 (44.3%)	Within 1 hour n (%) 2350 (55.7%)
**Child characteristics**		
***Sex of child***		
Male	1019 (54.1)	1201 (51.5)
Female	866 (45.9)	1132 (48.5)
***Size at birth***		
Larger than Average	953 (50.5)	1203 (51.6)
Average	602 (31.9)	783 (33.6)
Smaller than average	331 (17.6)	347 (14.9)
***Birth order***		
1	477 (25.2)	480 (20.6)
2–4	932 (49.4)	1267 (54.3)
5+	476 (25.2)	586 (25.1)
**Twin status**		
Single birth	1842 (97.7)	2271 (97.3)
Twin	44 (2.3)	62 (2.7)
***Type of assistance during delivery***		
Non-health professional	503 (26.7)	517 (22.2)
Health professional	1383 (73.3)	1816 (77.8)
***Place child was delivered***		
Home	502 (26.6)	536 (23.0)
Health facility	1384 (73.4)	1797 (77.0)
**Maternal factors**		
***Age***		
15–24	393 (20.9)	512 (22.0)
25–34	872 (46.2)	1143 (49.0)
35–49	621 (32.9)	678 (29.1)
***Marital status***		
Not Married	751 (39.8)	871 (37.3)
Married	1134 (60.2)	1463 (62.7)
***Religion***		
Christianity	1450 (76.9)	1774 (76.0)
Islam	292 (15.5)	415 (18.0)
Traditionalist	66 (3.5)	60 (2.6)
No region	78 (4.1)	85 (3.6)
***Ethnicity***		
Akan	860 (45.6)	1138 (48.8)
Ga-Dangbme	142 (7.6)	126 (5.4)
Ewe	279 (14.8)	282 (12.1)
Mole-Dagbani	305 (16.2)	426 (18.3)
Others	299 (15.9)	362 (15.5)
***Education***		
No education	426 (24.5)	644 (27.6)
Primary	406 (21.5)	422 (18.1)
Secondary	937 (49.7)	1151 (49.2)
Higher	80 (4.2)	116 (5.0)
***Working status***		
Not working	329 (17.5)	409 (17.5)
Working	1.556 (82.5)	1925 (82.5)
***Frequency of reading newspaper/magazine***		
Not at all	1655 (87.8)	2045 (87.7)
Less than once a week	150 (7.9)	145 (6.2)
At least once a week	81 (4.3)	143 (6.1)
***Frequency of listening to radio***		
Not at all	333 (17.7)	403 (17.3)
Less than once a week	600 (31.8)	780 (33.4)
At least once a week	952 (50.5)	1150 (49.3)
***Frequency of watching television***		
Not at all	538 (28.5)	667 (28.6)
Less than once a week	445 (23.6)	635 (27.2)
At least once a week	903 (47.9)	1032 (44.2)
***Number of ANC visits***		
0	58 (3.1)	51 (2.2)
1–3	215 (11.4)	198 (8.5)
4+	1613 (85.5)	2085 (89.4)
**Community/household factors**		
***Region***		
Western	176 (9.3)	260 (11.1)
Central	184 (9.8)	282 (12.1)
Greater Accra	330 (17.5)	353 (15.1)
Volta	172 (9.1)	151 (6.5)
Eastern	191 (10.1)	207 (8.9)
Ashanti	385 (20.4)	359 (15.4)
Brong-Ahafo	142 (7.5)	235 (10.1)
Northern	181 (9.6)	314 (13.4)
Upper east	58 (3.1)	124 (5.3)
Upper west	65 (3.4)	48 (2.1)
***Place of residence***		
Rural	1008 (53.5)	1269 (54.4)
Urban	878 (46.5)	1063 (45.6)
***Wealth index***		
Poor	765 (40.6)	984 (42.2)
Middle	403 (21.4)	442 (18.9)
Rich	717 (38.0)	907 (38.9)

Source: 2014 Ghana Demographic and Health Survey

### Statistical analysis

Analysis was done using Stata version 14. The first step of the analysis involved the distribution of early initiation of breastfeeding across the child, maternal and community/household level characteristics of women using frequency and percentages (see Table 1). This was followed by a bivariate analysis using logistic regression to assess the association between early initiation of breastfeeding and the child, maternal and community/household factors. This was presented in three models, with the child factors in model I, maternal factors in Model II and community/household factors in Model III. The results were presented as unadjusted odds ratios (UORs) at *p* < 0.05 (see Table 2). Variables that were significant at the bivariate level (5% margin), were used to build three models in a multivariable logistic regression analysis. Model I was made up of the child factors and early initiation of breastfeeding only. In Model II, the maternal factors only were added to the variables in Model I. Finally, Model III was a complete model that had the child, maternal and community/household factors and early initiation of breastfeeding. The results were presented as adjusted odds ratios (AORs) at p < 0.05 (see Table 3). Sample weight (v005/1000,000) was applied to correct for over-and under-sampling while the SVY command was used to account for the complex survey design and generalizability of the findings.

**Table 2:** Unadjusted Odds Ratio for early initiation of breastfeeding in Ghana (*n* = 4219)

Variables	Model I UOR (95%CI)	Model II UOR (95%CI)	Model III UOR (95%CI)
**Child factors**			
***Sex of child***			
Male	Ref		
Female	1.07 [0.94–1.21]		
***Size at birth***			
Larger than Average	Ref		
Average	0.98 [0.85–1.12]		
Smaller than average	0.89 [0.75–1.05]		
***Birth order***			
1	0.74^***^ [0.63–0.86]		
2–4	Ref		
5+	0.89 [0.77–1.51]		
**Twin status**			
Single birth	Ref		
Twin	1.03 [0.69–1.52]		
***Type of assistance during delivery***			
Non-health professional	0.79^***^ [0.69–0.91]		
Health professional	Ref		
***Place child was delivered***			
Home	0.82^**^ [0.72–0.94]		
Health facility	Ref		
**Maternal factors**			
***Age***			
15–24		0.97 [0.83–1.14]	
25–34		0.91 [0.79–1.05]	
35–49		Ref	
***Marital status***			
Not Married		0.98 [0.86–1.11]	
Married		Ref	
***Religion***			
Christianity		Ref	
Islam		1.11 [0.95–1.29]	
Traditionalist		0.66^*^ [0.48–0.91]	
No region		1.11 [0.81–1.52]	
***Ethnicity***			
Akan		Ref	
Ga-Dangbme		0.69^*^ [0.51–0.93]	
Ewe		0.73^**^[0.59–0.90]	
Mole-Dagbani		0.91 [0.78–1.06]	
Others		0.88 [0.75–1.05]	
***Education***			
No education		1.01 [0.88–1.16]	
Primary		0.91 [0.78–1.07]	
Secondary		Ref	
Higher		1.02 [0.74–1.41]	
***Working status***			
Not working		1.04 [0.89–1.22]	
Working		Ref	
**Media exposure**			
***Frequency of reading newspaper/magazine***			
Not at all		Ref	
Less than once a week		0.78 [0.61–1.01]	
At least once a week		1.39^*^ [1.02–1.90]	
***Frequency of listening to radio***			
Not at all		0.93 [0.79–1.10]	
Less than once a week		1.09 [0.95–1.25]	
At least once a week		Ref	
***Frequency of watching TV***			
Not at all		1.08 [0.94–1.24]	
Less than once a week		1.26^**^ [1.08–1.49]	
At least once a week		Ref	
***Number of ANC visits***			
0		0.74 [0.52–1.05]	
1–3		0.82 [0.67–1.00]	
4+		Ref	
**Community/household factors**			
***Region***			
Western			1.38^*^ [1.05–1.81]
Central			1.58^**^ [1.20–2.07]
Greater Accra			1.06 [0.80–1.42]
Volta			0.82 [0.61–1.09]
Eastern			1.05 [0.80–1.39]
Ashanti			Ref
Brong-Ahafo			1.74^***^ [1.33–2.28]
Northern			1.58^***^ [1.23–2.04]
Upper east			1.95^***^ [1.48–2.58]
Upper west			0.78 [0.59–1.04]
***Place of residence***			
Rural			Ref
Urban			0.97 [0.86–1.10]
***Wealth index***			
Poor			Ref
Middle			0.90 [0.77–1.06]
Rich			1.00 [0.87–1.15]

*p < 0.05, **p < 0.01, ***p < 0.001. *Ref* Reference category, *CI* Confidence Interval

Source: 2014 Ghana Demographic and Health Survey

### Ethical consideration

The DHS Program reports that ethics approval was sought from the National Public Health Reference Laboratory of the GHS and Noguchi Memorial Institute for Medical Research all in Ghana. It was further reported that informed consent was provided by all study participants.

## Results

### Distribution of early initiation of breastfeeding across the child, maternal and community/household characteristics of women

Table 1 presents results of the distribution of early initiation of breastfeeding across the child, maternal and community/household characteristics of women. We found that 55.7% of women initiated breastfeeding within the first hour of delivery. In terms of the child characteristics, most of the children who went through early initiation of breastfeeding were males (51.5%), larger than average size at birth (51.6%), 2–4 birth order (54.3%), were born single (97.3%), were delivered with the help of health professionals (77.8%), and at the health facility (77.0%). With the maternal factors, most of the women who practiced early initiation of breastfeeding were aged 25–34 (49.0%), married (62.7%), Christians (76.0%), Akans (48.8%), had secondary education (49.2%), never read newspaper (87.7%), listened to radio at least once a week (49.3%), watched television at least once a week (44.2), and had 4+ ANC visits. With community/household factors, women from the Ashanti region (15.4%), those in rural areas (54.4%) and those of poor wealth index had the highest prevalence of early initiation of breastfeeding.

### Bivariate results on the predictors of early initiation of breastfeeding in Ghana

Table 2 shows the bivariate results of early initiation of breastfeeding in Ghana. At *p* < 0.05, birth order (UOR = 0.74^***^ CI = [0.63–0.86]), place of delivery (UOR = 0.82^**^ CI = [0.72–0.94]) and type of assistance during delivery (UOR = 0.79^***^ CI = [0.69–0.91]) were the child factors that showed statistically significant relationship with early initiation of breastfeeding in Ghana. With maternal factors, religion (UOR = 0.66^*^ CI = [0.48–0.91]), ethnicity (UOR = 0.69^*^ CI = [0.51–0.93]), frequency of reading newspaper (UOR = 1.39^*^ CI = [1.02–1.90]) and frequency of watching television (UOR = 1.26^**^ CI = [1.08–1.49]) showed statistically significant associations with early initiation of breastfeeding at *p* < 0.05. Region was the only community/household variable that was significant at the bivariate level.

### Multivariable results

As shown in Table 3, children of first birth order [AOR = 0.71, CI = 0.61–0.84], those who were delivered by non-professionals [AOR = 0.51, CI = 0.30–0.88] and those whose mothers were Traditionalists [AOR = 0.65, CI = 0.46–0.92] and Mole-Dagbanis [AOR = 0.69, CI = 0.54–0.89] were less likely to go through early initiation of breastfeeding compared to those of 2–4 birth order, those who were delivered by health professionals, those whose mothers were Christians and Akans, respectively. Conversely, children born to mothers who read newspaper/magazine at least once a week were more likely to go through early initiation of breastfeeding compared to those who never read newspaper/magazine [AOR = 1.40, CI = 1.01–1.95]. Children born to mothers who watched television less than once a week were more likely to go through early initiation of breastfeeding compared to those who watched television at least once a week [AOR = 1.40, CI = 1.01–1.95]. Finally, women from the Northern [AOR = 2.40, CI = 1.77–3.26] and Upper East regions [AOR = 2.57, CI = 1.86–3.56] practiced early initiation of breastfeeding compared to those from the Ashanti region (see Table 3).

**Table 3:** Adjusted Odds Ratio for early initiation of breastfeeding in Ghana

Variable	Model I AOR (95%CI)	Model II AOR (95%CI)	Model III AOR (95%CI)
**Child factors**			
***Birth order***			
1st	0.718^***^[0.61–0.84]	0.72^***^[0.62–0.85]	0.71^***^[0.61–0.84]
2-4th	Ref	Ref	Ref
5 and above	0.93 [0.80–1.07]	0.911 [0.79–1.06]	0.91 [0.78–1.06]
***Type of delivery assistance***			
Non-health professional	0.50^*^[0.30–0.85]	0.53^*^[0.31–0.89]	0.51^*^[0.30–0.88]
Health professional	Ref	Ref	Ref
***Place of delivery***			
Health facility	1.55 [0.92–2.63]	1.47 [0.87–2.49]	1.45 [0.85–2.48]
Home	Ref	Ref	Ref
**Maternal factors**			
***Religion***			
Christianity		Ref	Ref
Islam		1.15 [0.96–1.37]	1.08 [0.89–1.30]
Traditionalist		0.72 [0.51–1.02]	0.65^*^[0.46, 0.92]
No region		1.16 [0.84–1.61]	1.21 [0.88–1.66]
***Ethnicity***			
Akan		Ref	Ref
Ga-Dangbme		0.71^*^[0.52–0.95]	0.83 [0.60–1.15]
Ewe		0.77^*^[0.63–0.95]	1.03 [0.78–1.35]
Mole-Dagbani		0.88 [0.73–1.05]	0.69^**^ [0.54–0.89]
Others		0.92 [0.76–1.11]	0.71^**^[0.56–0.90]
***Frequency of reading newspaper/magazine***			
Not at all		Ref	Ref
Less than once a week		0.84 [0.64–1.09]	0.86 [0.66–1.13]
At least once a week		1.44^*^[1.04–1.99]	1.40^*^ [1.01–1.95]
***Frequency Watching television***			
Not at all		1.19^*^ [1.02–1.39]	1.13 [0.96–1.32]
Less than once a week		1.30^**^ [1.11–1.54]	1.28^**^ [1.08–1.51]
At least once a week		Ref	Ref
**Household and community factors**			
***Region***			
Western			1.38^*^ [1.04–1.82]
Central			1.53^**^ [1.16–2.02]
Greater Accra			1.09 [0.80–1.48]
Volta			0.86 [0.60–1.22]
Eastern			1.09 [0.82–1.46]
Ashanti			Ref
Brong Ahafo			1.84^***^ [1.40–2.42]
Northern			2.40^***^[1.77–3.26]
Upper east			2.57^***^[1.86–3.56]
Upper west			1.09 [0.78–1.53]
pseudo *R*^2^	0.005	0.012	0.028
Hosmer-Lemoshow	8.66	397.35	1113.91
ROC Area	0.5466	0.5694	0.6091

*p < 0.05, **p < 0.01, ***p < 0.001. *Ref* Reference category, *CI* Confidence Interval

Source: 2014 Ghana Demographic and Health Survey

## Discussion

In this study, we investigated the determinants of early initiation of breastfeeding among child bearing women in Ghana. More than half (55.7%) of the surveyed women initiated breastfeeding within the first 1 hour of delivery. It was also found that birth order, type of assistance during delivery, religion, ethnicity, exposure to newspaper, television, and region are associated with early initiation of breastfeeding. The prevalence of early initiation of breastfeeding places Ghana above some low-and middle-income countries such as Nepal where a recent national survey reported 41.8% breastfeeding initiation within the first 1 hour of delivery [13]. Meanwhile, higher rates of early breastfeeding initiation have been reported from Ethiopia [1], Namibia [15], and Bangladesh [17]. Ghana falls below the WHO guidelines which recommends that all new-borns should be breastfed within the first 1 hour of birth [2]. Early initiation of breastfeeding can be enhanced if positive societal attitude and political will are marshalled to create enabling environment and support systems that will expedite initiation of breastfeeding within the first 1 hour after childbirth. Regulating the breastmilk substitute industries, initiating and establishing effective monitoring systems of early breastmilk initiation for all deliveries could improve the situation [18, 19].

We noted a significant association between the type of delivery assistance and early breastfeeding initiation. Women who were assisted by non-health professionals were less likely to initiate breastfeeding within the first hour of delivery. In Ghana, nearly all health professional assisted deliveries take place in health facilities whilst traditional birth attendants dominate in home deliveries [20]. This suggests that most of the women who were assisted by health professionals and initiated breastfeeding within the first hour of delivery gave birth in health facilities. Our observation echoes argument in literature about how obtaining health professional assistance at delivery enhances early breastfeeding initiation [15]. The finding underscores the established relevance of health professionals in sustaining and improving maternal and new-born health [21]. Maternity healthcare providers need to be constantly motivated and reminded through regular educational programmes, memos and reader-friendly audio-visuals in order for them to routinely educate women about the essence of early breastfeeding initiation [22].

Children of first birth order were less likely to have early breastfeeding initiation than those of 2–4 birth order. This finding is consistent with Ethiopia [23] and Nepal [13] based studies which found high occurrence of early initiation of breastfeeding among children who were born second or later compared with first borns. In the case of Nepal, most first borns were fed with prelacteal feeds (e.g. honey or ghutti) prior to breastfeeding [13]. Prelacteal feeds are alien to the Ghanaian culture but we do not doubt the possibility of its gradual permeation into the Ghanaian market and possible preference by primiparous women. These primiparous women might have not initiated early breastfeeding because of the fear that they might not have sufficient milk secretion to breastfeed as it is their first birth [4].

Women who listened to radio at least once a week were more likely to practice early breastfeeding initiation. However, women who watched television once a week had lower odds of early breastfeeding initiation. The benefit of media exposure to early breastfeeding initiation has been reported [23]. Our finding could imply that pro early breastfeeding initiation programmes/advertisements are channelled through radio than television as the former is common, mobile and easily accessible for all Ghanaians irrespective of wealth status or location [24]. The finding suggests that in low-and middle-income countries, utilising easily accessible media avenues could yield greater gains in increasing early breastfeeding initiation rate.

Some variations existed across the administrative regions such that women in Northern and Upper East regions had higher chances of early breastfeeding initiation. Geographically, these two regions share a common boundary and both lie in the northern sector of the country. This could imply possible favourable contextual factors within these parts of the country which are not empirically explored. It is worthy of mention that significant ecological variations exist between the northern and southern Ghana [25]. Variation in early breastfeeding initiation due to contextual factors such as agro-ecological zones has also been noted in Nepal [13]. There could be variation in advocacy and recommendation enforcement strategies utilised by health facilities within these locations [26, 27].

### Strengths and limitations

This study is a cross sectional survey thereby limiting causal inference. We admit the possibility of recall bias especially among multiparous women. Yet, its national representativeness and large sample enhance generalisability prospects to other low-and middle-income countries especially in Africa. In addition, we could not include other variables such as maternal nutrition status, cultural taboos, and health system related factors that were not captured in the DHS.

## Conclusion

Early initiation of breastfeeding is relatively low in Ghana in light of the WHO recommendation as not all newborns are breastfed within the first 1 hour of delivery. Children of first birth order, those who were delivered by non-professionals, those whose mothers were Traditionalists, and Mole-Dagbanis had lower chances of early breastfeeding initiation. Reading newspaper/ magazine, watching television were the factors that enhanced early breastfeeding initiation. Empowering healthcare providers to be consistent in early breastfeeding initiation advocacy and effective community engagement on the need to embrace and practice this can as well improve the situation. Factors contributing to early breastfeeding initiation in the Northern parts of Ghana can be investigated in order to scale up the protective measures to other parts of the country and other low-and middle-income African countries.

## Data Availability

All analysed data are freely available to the public from the Measure DHS website, thus www.measuredhs.org.

## Abbreviations

AOR: Adjusted Odds Ratio
ANC: Antenatal Care
CI: Confidence Interval
GDHS: Ghana Demographic and Health Survey
GHS: Ghana Health Service
WHO: World Health Organisation
